# The lunar dust environment: concerns for Moon-based astronomy

**DOI:** 10.1098/rsta.2023.0075

**Published:** 2024-05-09

**Authors:** Mihály Horányi, Jamey R. Szalay, Xu Wang

**Affiliations:** ^1^ Laboratory for Atmospheric and Space Physics, and Department of Physics, University of Colorado, Boulder, CO, USA; ^2^ Department of Astrophysical Sciences, Princeton University, Princeton, NJ, USA

**Keywords:** moon, dust hazard, near-surface dusty plasmas

## Abstract

The Moon has no atmosphere, hence, it offers a unique opportunity to place telescopes on its surface for astronomical observations. It is phase-locked with Earth, and its far side remains free from ground-based interference, enabling the optimal use of radio telescopes. However, the surface of the Moon, as any other airless planetary object in the solar system, is continually bombarded by interplanetary dust particles that cause impact damage and generate secondary ejecta particles that continually overturn the top layer of the lunar regolith. In addition, there is evidence, that small particles comprising the lunar regolith can be electrically charged, mobilized and transported, also representing a hazard for covering sensitive surfaces and interfering with exposed mechanical structures. In addition to the naturally occurring dust transport, rocket firings during landings and take-offs, pedestrian and motorized vehicle traffic will also liberate copious amounts of dust, representing a potential hazard for the safe and optimal use of optical platforms.

This article is part of a discussion meeting issue ‘Astronomy from the Moon: the next decades (part 2)’.

## 1. Introduction

The surface of the Moon is covered with a layer of loose rocky material, including micron and submicron-sized dust particles. This regolith has been formed and remains continually reworked, by the intermittent impacts of comets, asteroids, meteoroids and the continual bombardment by interplanetary dust particles (IDPs). All planetary bodies in the inner solar system are continually bombarded by IDPs originating primarily from asteroid collisions and cometary activity. Thick atmospheres protect Venus, Earth and Mars, ablating the incoming IDPs into ‘shooting stars’ that rarely reach the surface. On the contrary, the surface of airless planetary bodies are directly exposed to IDP impacts. The effects of meteoroids bombarding the lunar surface, especially their contribution to sustaining the tenuous lunar atmosphere, have been recognized since the Apollo era, but there are still large uncertainties in our knowledge about the variability of their flux, size and speed distributions [1]. In addition, the solar wind plasma flow and solar ultraviolet radiation also reach the surface generating a near-surface plasma environment that can lead to charging, mobilization and transport of the lunar fines.

Dust poses risks to human presence or the long-term remote operation of astronomical telescopes on the lunar surface. Dust particles damage spacesuits [2], cover optical surfaces [3–5] and degrade the performances of thermal radiators and solar panels [2]. Lunar dust in human living quarters could lead to health risks when inhaled by astronauts [6].

Below we discuss the effects of the IDP influx (§2), the properties of the secondary ejecta particles they generate (§3) and the near-surface plasma effects on the lunar regolith (§4), as these all represent a potential hazard for establishing permanent human habitats, and to use the unique opportunity the Moon offers to deploy astronomical observatories [7,8]. We offer our assessment for ranking the potential risk these processes represent (§5).

**Figure 1.**
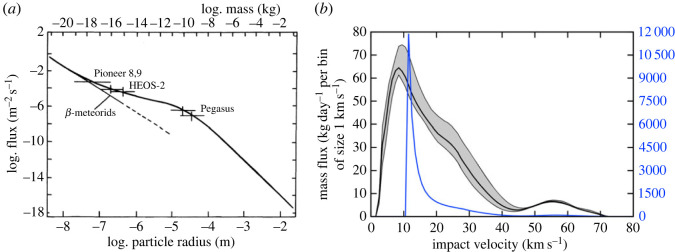
(*a*) The flux of interplanetary meteoroids at 1 AU as a function of their size (mass), the labels indicate space missions, and β-meteoroids are IDPs on escaping orbits driven by radiation pressure [9]; (*b*) the modelled speed distribution, independent of the size of a meteoroid, scaled with the mass flux at the Moon (black solid line) and at Earth (blue solid line) [11,12].

## 2. Interplanetary dust bombardment

The surfaces of airless bodies near 1 AU are directly exposed to high-speed ($\gg 1\;\text{km}\;{\text{s}}^{-1}$) micrometeoroid impacts, particles with characteristic radii of ≃100 μm dominating the mass flux of $F≃1.5\times 10^{-15}\;\text{kg}\;{\text{m}}^{-2}\;s^{-1}$ [9]. The most recent estimate of the cosmic dust input into the Earth’s atmosphere is (43±14) tons per day [10]. The mass flux of meteoroids impacting the lunar surface is about 30 times smaller due to the Moon’s smaller size and reduced gravitational focusing, resulting in an average total deposition rate of (1.4±0.5) tons per day [11]. IDPs arrive at the lunar surface with a characteristic speed of $≃10\;\text{km}\;{\text{s}}^{-1}$ [11]. Figure 1 shows the size, g(m), and speed, h(v), distributions that are assumed to be independent, hence f(m,v)≃g(m)×h(v). The IDP sources impacting the Moon at high latitudes remain largely uncharacterized due to the lack of optical and radar observations in the polar regions on Earth [13]. High-latitude sources could have very large impact speeds in the range of $30\leq v\leq 50\;\text{km}\;{\text{s}}^{-1}$ [14,15], hence they are expected to have a significant effect on the lunar surface, including the removal and burial of volatile deposits in the lunar polar regions.

### (a) Concern for lunar-based astronomy

IDP impacts will excavate craters on exposed surfaces. One of the goals of NASA’s Long Duration Exposure Facility mission (LDEF) was to survey the interplanetary and Earth-orbital dust populations [16]. LDEF was placed in low-Earth orbit (LEO) by the space shuttle Challenger in April 1984, and retrieved by the space shuttle Columbia in January 1990, orbiting for 5.77 years in the altitude range of 331–480 km. After retrieval, a set of 761 craters was identified on a $5.6\;m^{2}$ zenith-facing aluminium alloy panel, with diameters in the approximate range of 10 μm–1 mm [17]. Each micrometeoroid impact generates a dent, with a radius and depth set by its mass, speed and composition, degrading the performance of optical surfaces at an approximate rate of 0.01%/year at 1 AU distance from the Sun, with large possible fluctuations due to the stochastic nature of impacts. In the first approximately six months of operations, the Webb telescope’s primary mirror, with a total surface area of about $30\;m^{2}$, was hit five times, without generating any noticeable degradation in its performance [12]. During the predickted lifetime of 20 years, the Webb telescope’s primary mirror is expected to steadily accumulate damages that only minimally degrade its performance, neglecting additional damages by the rare and hard-to-predict larger (≫10 μm) IDPs. Similar degradation is expected for optical telescopes on the lunar surface.

## 3. High-altitude dust ejecta cloud

**Figure 2.**
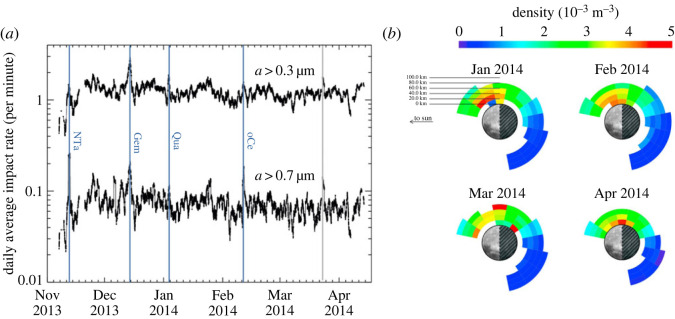
(*a*) The daily running average of impacts per minute of particles with radii > 0.3 µm and a>0.7 µm recorded by LDEX. Four annual meteoroid showers generated elevated impact rates lasting several days. The labelled annual meteor showers (blue vertical lines) are the Northern Taurids (NTa); the Geminids (Gem); the Quadrantids (Qua); and the Omicron Centaurids (oCe). Towards the end of March LDEX data indicated a meteor shower that remained unidentified by ground-based observers [19]. (*b*) The average dust ejecta cloud density observed by LDEX for each calendar month LADEE was operational in 2014. Each colour ring corresponds to the density every 20 km. The plot is in a reference frame where the Sun is on the left (−x direction) and the apex motion of the Moon about the Sun is towards the top of the page (+y direction) [21,22]. The missing bottom left quadrants represent data gaps, as LDEX could not make measurements while the Sun was in its FOV.

In addition to direct damage, IDP impacts generate secondary ejecta particles, lofting them to high altitudes (≫100 km), some even escaping the Moon. The bound ejecta particles form a permanently present dust cloud engulfing the Moon that was identified during NASA’s Lunar Atmosphere and Dust Environment Explorer (LADEE) mission [18]. LADEE was launched in September 2013, it reached the Moon in about 30 days, and continued with an instrument checkout period of about 40 days at an altitude range of 220–260 km, followed by approximately 150 days of science observations period at a typical altitude range of 20–100 km. LADEE followed a near-equatorial retrograde orbit, with a characteristic orbital speed of $1.6\;\text{km}\;{\text{s}}^{-1}$. It carried the Lunar Dust Experiment (LDEX) that was designed to explore the ejecta cloud generated by sporadic interplanetary dust impacts, including possible intermittent density enhancements during meteoroid showers, and to search for the putative regions with high densities of dust particles with radii ≪ 1 µm lofted above the terminators [19]. LDEX was an impact ionization dust detector that measured both the positive and negative charges of the plasma cloud generated when a dust particle struck its target. The amplitude and shape of the waveforms (signal versus time) recorded from each impact were used to estimate the mass of the dust particles. The instrument had a total sensitive area of $0.01\;m^{2}$, gradually decreasing to zero for particles arriving from outside its dust field-of-view (FOV) of $\pm 68^{\circ }$ off from the normal direction [20]. The measured fluxes indicated that the Moon is engulfed in a permanently present, but highly variable dust exosphere (figure 2). The density of the dust exosphere, compared with our best models of the incoming primary meteoroid flux, is lower than expected. Based on LDEX observations, the total ejecta production rate on the Moon is of the order of $18\;\text{t}\;{\text{day}}^{-1}$, indicating a mass yield $Y({\mathrm{mass}}_{\text{secondary}}/{\mathrm{mass}}_{\text{primary}})≃10$ [11], 2–3 orders of magnitude smaller than the icy Galilean moons of Jupiter [23].

### (a) Concern for lunar-based astronomy

The vast majority of the ejecta particles are launched into a narrow cone angle normal to the surface with speeds below the escape speed from the Moon ($2.4\;\text{km}\;{\text{s}}^{-1}$), hence following ballistic trajectories and returning to the surface [24]. LDEX observations enable us to estimate the average lunar dust ejecta density distribution above the Moon, and the rate at which this exospheric dust rains back onto the lunar surface. Near the equatorial plane, the burial rate is estimated to be approximately $40\;\text{μ}\text{m}\;{\mathrm{Myr}}^{-1}$ of lunar regolith, with a cumulative size distribution index of $\gamma =2.7,(n(>a)∼a^{-\gamma })$, that is redistributed due to meteoritic bombardment, a process that occurs predominantly on the lunar apex hemisphere [25]. This burial rate is rather modest; however, it does not include any contribution that initially slow particles, which did not rise to the orbital altitude of LADEE, might contribute.

Additional sources of damage are the fast secondary ejecta particles that have speeds above the lunar escape speed, generated at shallow angles [26]. They dominate the generation of micro craters with diameters below 7 μm observed on lunar rocks [27,28]. The cratering rate of these small pits, compared with the predictions based on *in situ* measurements of micrometeoroids in space, indicate a flux of fast ejecta $≃10\;m^{-2}\;s^{-1}$ of particles with radii ∼1 μm [9], that generates a negligibly slow surface degradation rate due to cratering. However, high-speed hits can generate significant impact charges and electromagnetic pulses and can destroy sensitive electronics [29].

## 4. Near-surface dust mobilization

Compared with direct IDP impacts (§2), or impacts/burial by secondary ejecta particles (§3), perhaps the biggest concern for Moon-based astronomy is electrostatic dust charging, mobilization and transport, which is expected to dominate the near surface (≪1 km) dust environment [30,31]. The Moon has no global magnetic field and only a tenuous exosphere, hence, its surface is directly exposed to the solar wind plasma flow, the Earth’s magnetospheric plasma environment and solar ultraviolet (UV) radiation, resulting in electric charging of the regolith that varies in space and time [32]. Electrostatic charges are expected to accumulate on human and robotic exploration systems, causing potential issues for crew safety and for the performance of scientific instruments and equipment [33–36].

**Figure 3.**
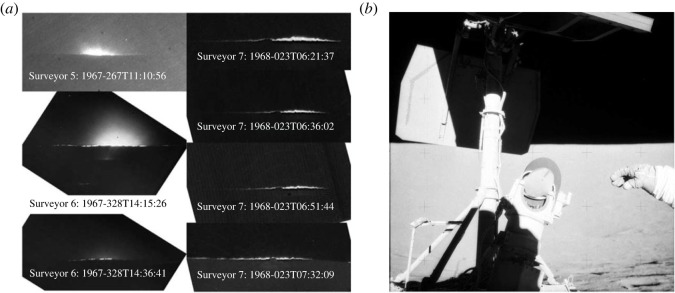
(*a*) Images of lunar horizon glow. The contribution of Zodiacal light is evident in Surveyor 5 and 6 but not in Surveyor 7 images, perhaps because of the different camera iris settings. (*b*) Apollo 12 Charles ‘Pete’ Conrad Jr. gestures near the Surveyor 3 spacecraft on the lunar surface on 21 November 1969. The Surveyor 3 television mirror shows a finger mark made by Conrad in a layer of dust on the mirror. Surveyor 3 landed on the Moon on 20 April 1967. Dust was likely deposited on the mirror both during the landing of the Surveyor and also during the landing of the Apollo 12 Lunar Module 155 m away (photos are from NASA’s Science Data Center).

Electrostatic processes on the lunar surface were first indicated by Apollo-era observations. The Lunar Horizon Glow (LHG), 30 cm above the surface was recorded shortly after sunset by the Surveyor landers (figure 3). The LHG is likely due to sunlight scattered off a cloud of dust particles with radii of a few µm that are lofted by electrostatic forces near the terminator [37–40]. The height of the LHG is consistent with a recent observation by the Chang’E-3 rover of fine dust deposits on lunar rocks up to a height of ≃30 cm [41].

The unexpected signals of the Apollo 17 Lunar Ejecta and Meteorites Experiment (LEAM) deployed on the lunar surface have been suggested to be due to low-speed $\left(<100\;\text{m}\;{\text{s}}^{-1}\right)$, highly charged dust particles with the rates spiking near sunset and sunrise [42]. However, different LEAM datasets showed no rate enhancement associated with terminator crossings and indicated that any enhancements were likely caused by rapid temperature changes rather than lofted dust [43]. Similarly, high-altitude dust possibly lofted through electrostatic mechanisms indicated from the Apollo astronauts’ sketches [44,45] and images from orbit [46,47] remained controversial. Such a dust population was not confirmed by the remote sensing observations by Clementine [48] and LRO/LAMP [49], or by the *in situ* measurements from LADEE/LDEX [21,50], contrary to a competing analysis [51]. These putative near-surface dust mobilizations might represent only modest dust fluxes, as opposed to dust mobilization due to human activities (figure 3), but they are acting continually for long periods of time, and without easy mission design solutions to mitigate their effects.

**Figure 4.**
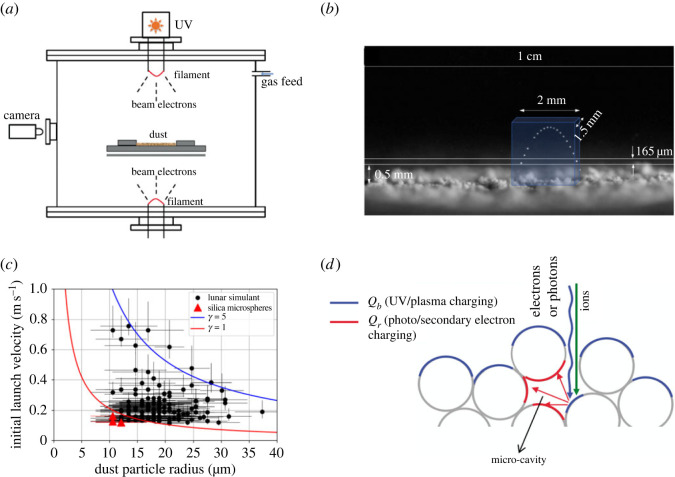
(*a*) The schematic of the experimental set-up to investigate dust charging, mobilization and transport under UV and/or plasma exposure of a regolith surface [59–62]. (*b*) Stacked images of the trajectory of a single dust particle lofted from the surface, using a narrow focal plane normal to the boresight of the camera. Images like this are used to measure the initial speed of the particle [62]. (*c*) Initial launch velocities as a function of dust size for irregularly shaped lunar simulant particles (circles) and 10 μm radius silica microspheres (triangles). The theoretical curves (solid lines), obtained from energy conservation, are shown with γ=1 and 5 that parameterize the cohesion between particles [62]. (*d*) Cartoon of the ‘patched charge model’ [59].

Through the decades following the Apollo missions, the processes responsible for the initial lift-off of dust particles remained poorly understood, even though a number of models [52–54] and laboratory experiments [55–58] were dedicated to this issue and succeeded in explaining the subsequent dynamics of charged dust particles in plasma sheaths. Recently, the recognition of the roles micro-cavities play in surface charging led to a better understanding of particle lift-off from surfaces due to plasma effects, the so-called patched charge model [59]. The model, verified by a series of experiments (figure 4) [60–66], is based on the emission and re-absorption of secondary electrons, generated by impacting energetic (≫1 eV) electrons, ions or UV photons. As opposed to the case of a flat surface, secondary electrons can remain trapped in cavities that form between dust particles, generating a potential difference of the order of 2–3 V, due to the typical energy of secondary electrons. This potential difference for a photoelectron plasma sheath on the Moon, with a daytime characteristic shielding thickness of the order of 1 m, generates an electric field ≃2 to $3\;\text{V}\;{\text{m}}^{-1}$. In a cavity, the same potential difference develops across characteristic distances of perhaps 10 to 100 μm, generating surprisingly large electric fields of the order of $10\text{–}100\;\text{kV}\;{\text{m}}^{-1}$, accompanied by highly enhanced, both positive and negative surface charge density distributions. The combination of complex electric fields and charge density distributions leads to ‘Coulomb explosions’, which break the cohesive forces between dust particles, and lead to the initial lift-off of charged grains (figure 4) [59]. Contrary to the expectations of positively charged particles, the emergence of highly negatively charged particles from a rough surface under UV illumination has been verified in laboratory experiments [60] and successfully explained using computer simulations [67].

### (a) Concern for lunar-based astronomy

The lunar soil samples, returned by the Apollo missions, have a size range of 1 μm to 1 cm and an approximately log-normal size distribution with a typical maximum in the range of 60–120 μm [68,69]. The small optical dust detectors on Apollo 12, 14 and 15 [70], each placed 1 m above the surface, indicated a combined long-term dust accumulation rate of the order of $100\;\text{μ}\text{g}\;{\text{cm}}^{-2}\;y^{-1}$ [71]. The Chang’E-3 lander’s Sticky Quartz Crystal Microbalance (SQCM), at a height of 1.9 m above the lunar surface, reported a dust accumulation rate of about $20\;\text{μ}\text{g}\;{\text{cm}}^{-2}\;y^{-1}$ [72]. Without knowing the initial speed and velocity distribution of the dust grains launched from the surface, it remains an open question how the dust accumulation rate changes with height above the surface. Assuming that these were similar at all the Apollo and the ChangE’3 landing sites, and using the observations at these two distinct heights to determine an approximate scale height, assuming that $\dot{m}={\dot{m}}_{0}\exp(-h/h_{0})$ for dust deposition, indicates $h_{0}≃0.6\;\text{m}$, and an accumulation rate $\dot{m_{0}}=500\;\text{μ}\text{g}\;{\text{cm}}^{-2}\;y^{-1}$ on the surface. If the characteristic radius of the mobilized dust particles is a=10 μm, the coverage of an exposed flat surface (h=0) by dust is about 15%/y, if a = 50 μm the coverage is 3%/y, and if a=100 μm, the coverage reduces to 1.5%/y.

The size and initial speed distributions of lofted particles were measured in laboratory experiments (figure 4), establishing an expected lofting rate, if extrapolated to lunar conditions, of 1–$10\;{\mathrm{cm}}^{-2}\;s^{-1}$ [61]. While in the tabletop laboratory experiments this initial rate dropped in minutes due to a lack of reciprocal dust transport in/out of the small crater holding the lunar simulant, it is expected that the dust lofting rates on the lunar surface could be sustained over geological timescales. The typical lift-off speed of $≃0.5\;\text{m}\;{\text{s}}^{-1}$ in these experiments indicates that the particles in the typical size of 10 to 20 μm could reach heights of > 10 cm on the lunar surface. If 10 μm particles are lofted at a rate of $1\;{\mathrm{cm}}^{-2}\;s^{-1}$ they generate a surface coverage rate of nearly 30% per day of landing particles. Some of the deposited grains could be subsequently removed by the very same processes, but dust mobilization from a smooth surface covered by a single layer of dust is expected to be much less efficient. The laboratory experiments cannot possibly reproduce lunar vacuum, UV radiation and plasma conditions, or the properties of the regolith, hence, the efficacy of plasma and UV-induced dust charging, mobilization and transport on the lunar surface remains an open issue. It is perhaps still encouraging that the rough estimates based on the combined Apollo [71] and ChangE’3 [72] *in situ* measurements are of similar magnitude as the laboratory results [61]. These processes could represent a significantly large enough hazard to warrant a combination of *in situ* measurements.

## 5. Conclusion

Dust on the lunar surface represents a variety of hazards and its safe and effective mitigation requires detailed, yet to-be-fully developed, engineering approaches. The lunar dust is extremely abrasive, similar to broken glass, it ruins friction-bearing surfaces, seals, gaskets, optical lenses, windows, degrades thermal radiators, solar panels and causes dangerous physiological effects on the tissue in human lungs [2].

Solar panels and optical devices on the lunar surface will lose performance due to the accumulation of dust particles on their surfaces. However, it is possible that dust accumulation can reach equilibrium due to the accompanying processes that can lead to concurrent dust removal. The Apollo Lunar Laser Retroreflectors deployed during Apollo 11, 14 and 15 are still operating after well over 40 years; however, the magnitude of the return signal has decreased by a factor of 10 to 100 since the arrays were deployed [3–5]. Even at the very start of their use, the strength of the return signal was about 10% of the expected value, based upon an analysis of the ground stations and the retroreflector arrays. A deposit of lunar dust on the front faces of the reflectors is the most likely reason, other causes have also been suggested, for example, the darkening of the glass material due to UV and/or particle exposure, micrometeoroid bombardment, or change in the thermal properties due to dust, UV and or plasma exposure. The dust may be due to secondary ejecta from micrometeorite impacts in the vicinity, electrically levitated dust and/or dust from the lift-off of the Apollo lunar module [4].

Most open questions about near-surface dust mobilization could be answered by precursor missions delivering small dedicated experiments to the lunar surface. For example, the size and speed distributions of electrostatically levitated dust could be measured on the surface [73,74]. A complementary measurement of the dust coverage as a function of height could be measured by the optical transmission changes of a series of glass plates [75] or quartz crystal microbalance set-ups [72].

We focused on the naturally occurring dust hazards, but rocket firings during landings and take-offs, pedestrian and motorized vehicle traffic, for example, will liberate copious amounts of dust, representing a potential hazard for the safe and optimal use of optical platforms [76]. These, however, could be mitigated by careful mission design using distant landing/take-off sites, minimizing any traffic near installations and by including shutters and covers over sensitive surfaces that can be deployed during critical periods, as needed. Site selections for the various scientific installations, and their long-term optimal use, will require international agreements and cooperations [77].

## Data Availability

This article has no additional data.
